# What Technological and Economic Elements Must be Addressed to Support the Affordability, Accessibility, and Desirability of Alternative Proteins in LMIC?

**DOI:** 10.1016/j.cdnut.2023.102027

**Published:** 2023-10-31

**Authors:** Katrin Gradl, Ana Sofía Sánchez Hernández, Warren L. Grayson, Tim JA. Finnigan, Hannah E. Theobald, Bahman Kashi, Veronika Somoza

**Affiliations:** 1 Leibniz-Institute for Food Systems Biology at the Technical University of Munich, Freising, Germany; 2 Department of Biotechnology Engineering, School of Engineering, Tecnológico de Monterrey, Monterrey, N.L., Mexico; 3 Department of Biomedical Engineering, Johns Hopkins University School of Medicine, Baltimore, MD, United States; 4 Marlow Foods Limited, Station Road, Stokesley, United Kingdom; 5 Economic Evaluation and Research, Limestone Analytics, Kingston, Ontario, Canada; 6 Department of Economics, Queen's University, Kingston, Ontario, Canada; 7 Department of Physiological Chemistry, Faculty of Chemistry, University of Vienna, Vienna, Austria; 8 Chair of Nutritional Systems Biology, TUM School of Life Sciences, Technical University of Munich, Freising, Germany

**Keywords:** alternative protein, LMIC, malnutrition, taste, protein technological processing, plant-based protein, fermentation, mycoprotein, cellular agriculture, cultivated meat

## Abstract

Populations in low- and middle-income countries (LMIC) typically consume less than the recommended daily amount of protein. Alternative protein (AP) sources could help combat malnutrition, but this requires careful consideration of elements needed to further establish AP products in LMIC. Key considerations include technological, nutritional, safety, social, and economic challenges. This perspective analyzes these considerations in achieving dietary diversity in LMIC, using a combination of traditional and novel protein sources with high nutritional value, namely, soy, mycoprotein, and cultivated meat. Technological approaches to modulate the technofunctionality and bitter off-tastes of plant-sourced proteins facilitate processing and ensure consumer acceptance. Economic considerations for inputs, infrastructure for production, and transportation represent key elements to scale up AP. Dietary diversification is indispensable and LMIC cannot rely on plant proteins alone to provide adequate protein intake sustainably. Investments in infrastructure and innovation are urgently needed to offer diverse sources of protein in LMIC.

## Introduction

Many communities in low- and middle-income countries (LMIC) suffer from the double burden of malnutrition, the coexistence of under- and overnutrition in the same individual and within the same community. Globally, food systems in LMIC often do not ensure the availability of diverse diets for all population groups, resulting in deficiencies of essential micronutrients needed for health and productivity [1]. Diets in LMIC tend to be staple grain-based and lack diversity, resulting in protein and micronutrient malnutrition [2]. A large part of the population consumes protein below the recommended daily allowance [3]. Signs of low protein intake may include growth failure, weak muscles, edema, thin and brittle hair, skin lesions, and biochemical changes, such as low serum albumin and hormonal imbalances [3].

Essential amino acids in dietary protein are typically more concentrated and bioavailable in animal-sourced foods than in nonanimal-sourced foods [4]. Given the negative environmental impact of conventional animal production, contributing to climate change, as well as health concerns from overconsumption [2], whether to consume animal-sourced foods has stimulated debates worldwide. Because amino acid fortification and supplementation programs are not widely used in LMIC [1], alternative protein (AP) options could support the adequate intake of essential amino acids to mitigate malnutrition.

Conventional methods of protein production have a high environmental impact, namely, factory farming of livestock [5]. However, smallholder farming of livestock supports livelihoods and nutritional needs in LMIC [6]. Through more efficient use of natural resources, desirable sensory attributes, affordable price, and high nutritional values, when fortified, AP presents a valuable protein source in addition to traditional animal-sourced proteins. This perspective evaluates the technical and economic factors to consider for establishing 3 AP biotechnology sectors and products, including plant-based, fermentation-derived, and cultivated, in LMIC.

## Protein Quality and Sources

Animal and plant proteins consist of amino acids, of which the proportion varies as a characteristic of the originating source. The human body does not synthesize 9 amino acids, referred to as indispensable amino acids (IAA). The content and availability of IAA determine the nutritional quality of dietary proteins, as they are essential for the growth, reproduction, and maintenance of the human body [3,5]. The Food and Agricultural Organization (FAO) developed an approach to quantify protein quality called Protein Digestibility-Corrected Amino Acid Score (PDCAAS) [4]. PDCAAS corrects the IAA for the digestibility of the dietary protein. In Table 1 [7,8], the PDCAAS values for selected foods from animal and plant sources are listed. Only soy protein and mycoproteins are comparable to animal protein sources concerning their contents and digestibility of essential amino acids. However, the FAO proposed to replace the PDCAAS with a new method called Digestible Indispensable Amino Acid Score (DIAAS). The DIAAS accounts for the ileal amino acid digestibility and does not include microbial metabolization in the large intestines [9].

**TABLE 1:** Values for Protein Digestibility-Corrected Amino Acid Score (PDCAAS), energy content, and major nutrients for selected animal and plant protein sources per 100 g product [3,7,8]

Product	PDCAAS	Protein (g)	Fibers (g)	Carbohydrates (g)	Fats (g)	Calories (kcal)
Casein	1	81.5			0.8	362
Egg white	1	11.1		0.7	0.03	47
Chicken	0.95	18.2			11.2	173
Beef	0.92	22.5			4.5	130
Mycoprotein	0.99	13	7.5	2.3	1.7	92
Soy protein concentrate	0.99	80	5	7	3	338
Soy	0.91	34.9	22	6.3	18.3	329
Beyond meat	0.88	15	2.1	4.8	17	238
Peas	0.68	22.9	16.6	41.2	1.4	271
Tofu	0.56	8.1		1.9	4.8	83
Lentils	0.52	23.4	17	40.6	1.6	270
Maize	0.52	8	9.7	64.2	3.8	323
Groundnuts	0.52	25.3	11.7	7.5	48.1	564
Rice	0.5	6.8	1.4	77.7	0.6	344
Wheat	0.4	10.6	13.3	59.6	1.8	297

PDCAAS, Protein Digestibility-Corrected Amino Acid Score.

### Plant-based proteins, products, and processing technology

The majority of agriculturally produced edible plant protein is used as animal feed [10]. However, the production of plant-based foods for human nutrition is more efficient, uses fewer natural resources, and reduces environmental impact [11]. Furthermore, 4 crops, including soy, wheat, corn, and rice, dominate the current global production of human edible plant protein [5]. Table 1 gives an overview of the nutritional value of selected animal and plant protein sources. Soy contains a comparatively high amount of protein (34.9 g protein per 100 g) and demonstrates the highest nutritional value of plant-based proteins. Cereals such as wheat, corn, and rice are of lower protein content (10.6, 8.0, and 7.2 g per 100 g, respectively [7]), and are more limited in their essential amino acid contents than legumes and nuts. However, beans and nuts are generally low in methionine, whereas corn is low in tryptophan, whereas grains are low in lysine. In general, plant proteins contain higher amounts of nonessential amino acids, such as arginine, glycine, alanine, and serine, demonstrating the need to diversify protein sources for human nutrition [10].

Protein-rich vegetables such as pulses, nuts, and seeds are already traditional components of diets in many developing and emerging countries. Promoting the cultivation and use of these plants could support cost-effective diets, reduce the need for more expensive imported protein sources, and strengthen local agriculture. So far, the main crop used in alternative plant-based products is soy, followed by wheat, and pea [10]. To use these plant-based protein alternatives in food formulations, various technofunctional and sensorial challenges must be resolved. Not all plant-based proteins have the same functionalities in their native states. Grain proteins, for example, demonstrate limited water solubility, whereas legume proteins typically form weak gels [12]. To mitigate these challenges, an adaptation of technological processing using physical, chemical, or biological methods is necessary. For instance, applying different temperatures, hydrostatic pressures, pH conditions, electromagnetic fields, hydration conditions or covalent, noncovalent, or hydrophobic fields, as well as enzymatic hydrolysis, are applied to improve the technofunctional properties of the protein [12]. Enzymatic fermentation has been recognized as a reasonable strategy to improve the bioavailability of minerals. Fermentation was reported to greatly reduce antinutrients such as phytic acid, helping to increase mineral bioavailability (ie, Ca, Zn, and Fe) [12]. Generally, enzymatic modification, resulting in protein hydrolysates, is preferred over chemical modification because it has faster reaction times, milder reaction conditions, and, in addition, can be designed more flexibly due to the specificity of the enzymes [12].

Plant proteins, hydrolysates, isolates, or concentrates thereof often exhibit a bitter taste and astringency, which limits their dietary use and may require the addition of taste-masking additives. The bitter taste of protein hydrolysates derives from peptides of predominantly hydrophobic amino acids [13], which are released during enzymatic or acidic hydrolysis and target oral bitter taste receptors after consumption. In addition to bitter peptides, a wide variety of other bitter compounds may also be present in protein isolates and concentrates. Examples of these include divalent ions, polyphenols, heterocycles, and alkaloids [12]. Because the presence of bitter peptides and other bitter compounds is limiting the technofunctionality of many plant-based proteins, various attempts have been made to prevent the formation of bitter-tasting compounds or mask their bitterness [14]. The most cost-effective, as well as technologically least complex method and therefore most suitable for LMIC, is masking the bitter taste by the addition of sweeteners. Flavor synergies can result from the interactions between bitter, salty, sour, and sweet compounds during formulation to suppress the transmission of bitter taste at a molecular level [14]. The use of strong flavors and sweeteners makes the bitter compound a component of a complex composition with multiple flavors, where the other nonbitter and pleasant flavors dominate. Using a combination of flavors and sweeteners offers advantages as they can act on different groups of taste receptors and therefore increase the sensory acceptability of plant protein hydrolysates used in plant-based AP foods. The biggest challenge to bringing APs to market in LMIC is cost. Significant upfront investments are required to build fermentation and product facilities to scale production, improve production efficiency, and reduce costs. Technological advances such as cheaper growth media or improved microflora strains could also contribute to price reductions [15].

### Mycoproteins, products, and technology

Mycoprotein is an AP source derived from fungi such as *Fusarium venenatum*, *Fusarium flavolapis*, *Rhizopus* spp, and *Neurospora* spp [16]. First developed as a food source in 1985, mycoproteins gained attention as a nutritious, environmentally conscious protein source [17]. Mycoprotein contains all 9 essential amino acids, vitamins, minerals, fiber (chitin [*N*-acetylglucosamine] and β-glucan [1,3-glucan and 1,6-glucan]) and has low fat, sugar, and sodium content. Mycoproteins also have a low carbon and water footprint. Mince products from Quorn, a meat substitute brand founded in the United Kingdom, create only 48% of the global warming potential by traditional animal protein on average [18] and only 4% of the beef equivalent [19]. Tofu, as an example of a plant-based AP, causes only 12% of the average carbon footprint of chicken and only 2% of beef [19]. Typically, dietary fiber in fungi is located in the cell wall as a complex structure composed of β-glucan, chitin, and mannoproteins. Recent findings from clinical trials demonstrated serum cholesterol-lowering effects from diets rich in mycoprotein, hypothesized to be the result of soluble mycoprotein fiber, which promotes the formation of short-chain fatty acids by the intestinal microbiota [20]. Because beef and chicken contain little dietary fiber, replacement with or integration of mycoprotein in the diet of emerging economies can increase average fiber consumption [21].

#### Technological considerations of mycoprotein fermentation

Major technological requirements for mycoprotein production include fermentation, harvesting, and mixing [16]. First, the fungal organism is grown in a continuous, sterilized fermentation system with carbohydrates, water, nitrogen, and other nutrients. Next, the fungus mycelium is heat-treated to reduce the ribonucleic acid content, suspended solids are centrifuged, and the biomass is recovered as a mycoprotein paste. Mycoprotein can be mixed with ingredients such as egg albumen, malt extract, and calcium which, along with cooking and freezing, creates changes in the rheology (material flow and deformation) to achieve a meat-like texture [16].

Federal investment in fermentation technology optimization and infrastructure construction could further expand mycoprotein production [16]. Key areas of improvement include innovations to fermentation bioreactors, RNA reduction with minimum biomass concentration loss, and adequate mixing of food texturizing agents with mycoprotein [16]. Moreover, converting carbohydrates from agricultural waste into additional mycoprotein fermentation sources, as was realized by Upcraft et al. [22] who successfully converted rice straw lignocellulose into fermentable substrate [23], could duly combat malnutrition and climate change [21].

#### Economic considerations of mycoprotein fermentation

Although the investments in the fermentation industry increased to US$1.7 billion in 2021, approximately US$1.5 billion of investment was concentrated in the North American region [24]. It is challenging to access fermentation facilities without funding because 80% of fermentation volumetric capacity is already contracted, and only one-sixth of the remaining capacity has processing capabilities for food production [24]. Even contracted fermentation capacity needs further investment for repair, as 95% of them were not designed for food production [24], which creates a barrier to local production of mycoprotein in LMIC.

Mycoprotein is currently a niche, expensive protein product, but the price might fall if production is scaled up by funding and fermentation facility provision [21]. Considerations about manufacturing facility installation and product transport are crucial in LMIC, as this would reduce shipping costs and economic burdens significantly [25].

Another economic consideration is that mycoprotein needs to maintain a comparable price with commercial meat or become a differentiated product. As per retail costs from the UK Tesco supermarket chain (March 2023), although steak mince and chicken breast fillets cost US$ 13.05 and US$ 7.97/kg, respectively, Quorn Mince costs US$ 10.36 for the same amount [8]. Further research into the costly extraction/dewatering process [16], prevention of fungi colonial mutants that interfere with meat texture and fermentation effectiveness, and local production of mycoproteins to resolve supply chain issues may help reduce costs [26]. To circumvent the problem of lack of cold chains, it is possible to dry mycoprotein to produce a powdered, protein-rich material or to process the mycoprotein products through high-temperature, short-term heat processing technologies.

#### Mycoprotein desirability

Consumer acceptance for mycoprotein primarily comes from healthiness, nutritional, and environmental benefits, although some consumers still have concerns about the sensory properties, mainly texture and taste [27,28]. However, continued improvements are leading to increased mycoprotein desirability. In terms of taste, different natural flavoring additives to mycoproteins can accommodate more consumers from high-income countries (HIC) and LMIC, as some consumers with plant-based diets prefer tastes diverging from traditional meat [27].

### Cultivated meat and technology

Cellular agriculture, a modern application of tissue engineering, supports the growth of a variety of animal cells for human consumption (ie, cultivated meat and seafood), without needing to slaughter animals [29]. According to the Good Food Institute, if produced at scale using renewable energy, cultivated meat is expected to have a 92% lower carbon footprint, use 95% less land, be almost 16 times more efficient at converting feed into meat, and use nearly 5 times less fresh water than conventional beef [30]. Cultivated meat products can have nearly identical and even tailored properties (eg, decreased fats, greater concentration of micronutrients) compared with conventional meat. However, barriers exist for establishing cultivated meat as a protein source in LMIC.

#### Cultivated meat technological and economic opportunities and challenges

Growth media, bioreactors, and electricity are the greatest technological cost drivers for cultivated meat. Optimized bioreactor designs are needed for improved oxygen transfer, minimal cell damage due to flow-induced shear forces [31], and reduced energy consumption. A large-scale (45,700 kg monthly output) cultivated meat production plant is projected to require a 450 million US$ investment, 10,000 to 100 orders higher than benchmark values for comparable traditional meat products [30]. Therefore, the development of more affordable, accessible equipment is still a needed factor for cellular agriculture to align with traditional methods, especially for LMIC markets.

Within growth media, only 2 growth hormones (FGF2 and TGF-β) and 2 recombinant proteins (insulin and transferrin) account for more than 90% of its cost [30]. In addition to recycling, innovations to reduce the cost of the growth medium include targeted delivery by tethering to biomaterial scaffolds to increase the efficiency of growth factor use by up to 90% [32] and machine learning–based discovery of medium components [33]. Supply of amino acids for large-scale production is important to consider, too, given that the current production could not fulfill the demand for industrial-scale cultivated meat [31].

The use of renewable energy in production plants will be required to make cultivated meat sustainable. Even so, renewable sources of energy still require significant subsidy to become viable and therefore bringing this technology to scale will require further accessibility innovations. Advancements in cryopreservation, cooling, and heating processes will play a key role in energy efficiency for cellular agriculture in LMIC and globally.

#### Desirability and LMIC market development of cultivated meat

Techniques to improve the flavor, aroma, and texture of cultivated meat may make it more appealing to consumers. These include the addition of cultured adipocytes, fermented lipid molecules, or oleaginous yeast to modulate fat profiles [34]. In the long term, the cellular agriculture industry could create tailored products with enhanced nutritional characteristics, which could be of special interest to LMIC communities experiencing nutritional deficiencies, provided retail prices pair those of current meat products.

The first challenge for market development in LMIC, however, is overcoming the lack of representation in the cellular agriculture industry. Considering the limited media coverage of this technology in LMIC, generating demand through consumer awareness and education campaigns on cellular agriculture must be one of the first steps in adopting cultivated meat in these countries [15].

## Culture, Social, and Safety Considerations for Diverse Alternative Proteins in Low- and Middle-Income Countries

A fundamental factor to consider in adopting AP in LMIC is current sources for protein consumption and demand for AP. LMIC such as South Africa, Argentina, China, and Brazil are among the largest LMIC meat consumers. However, LMIC consumers in Africa typically have a higher consumption of staple grains, whereas animal protein consumption only consists of 20%–30% of the total protein consumption [2].

In terms of consumer acceptance of cultivated and plant-based meat, 60% and 64% of educated Chinese consumers were found to be very likely to purchase these respective products. Acceptance was marginally lower for Indian consumers with 56% and 63% of consumers being very likely to purchase [35]. Plant-based AP is currently the most widely accepted AP source, and its popularity has grown steadily [2]. Mycoproteins and cultivated meat are relatively new AP sources still under development and may take time to gain widespread acceptance. Mycoprotein was granted Novel Food Status in the United Kingdom in 1983 and has been considered a Generally Recognized As Safe product by the Food and Drug Administration since 2002 [18], and plant-based products are available all over the world. Regulation of cultivated meat for consumption is necessary due to contamination possibilities throughout the manufacturing process [29].

Infrastructure challenges remain a barrier to increasing AP production in LMIC. If production plants for AP expand to LMIC, challenges will include the supply of reliable electricity and a sufficient workforce trained in specific biotechnological operations, as well as social and ethical issues.

The AP adoption must also give careful consideration to ensure this transition does not adversely affect people depending on livestock production [6]. Moreover, because in LMIC, the consumption of iron, vitamins, and essential lipids is lower than in HIC [21], AP products for LMIC must be comparable with meat in terms of nutrient intake. Fortification of micronutrients in food products can be an option [36]. Balancing the nutrients and integrating multiple protein sources would be especially crucial so that the consumption of a product can create a diverse nutritious diet [21].

## Conclusion

**FIGURE 1:**
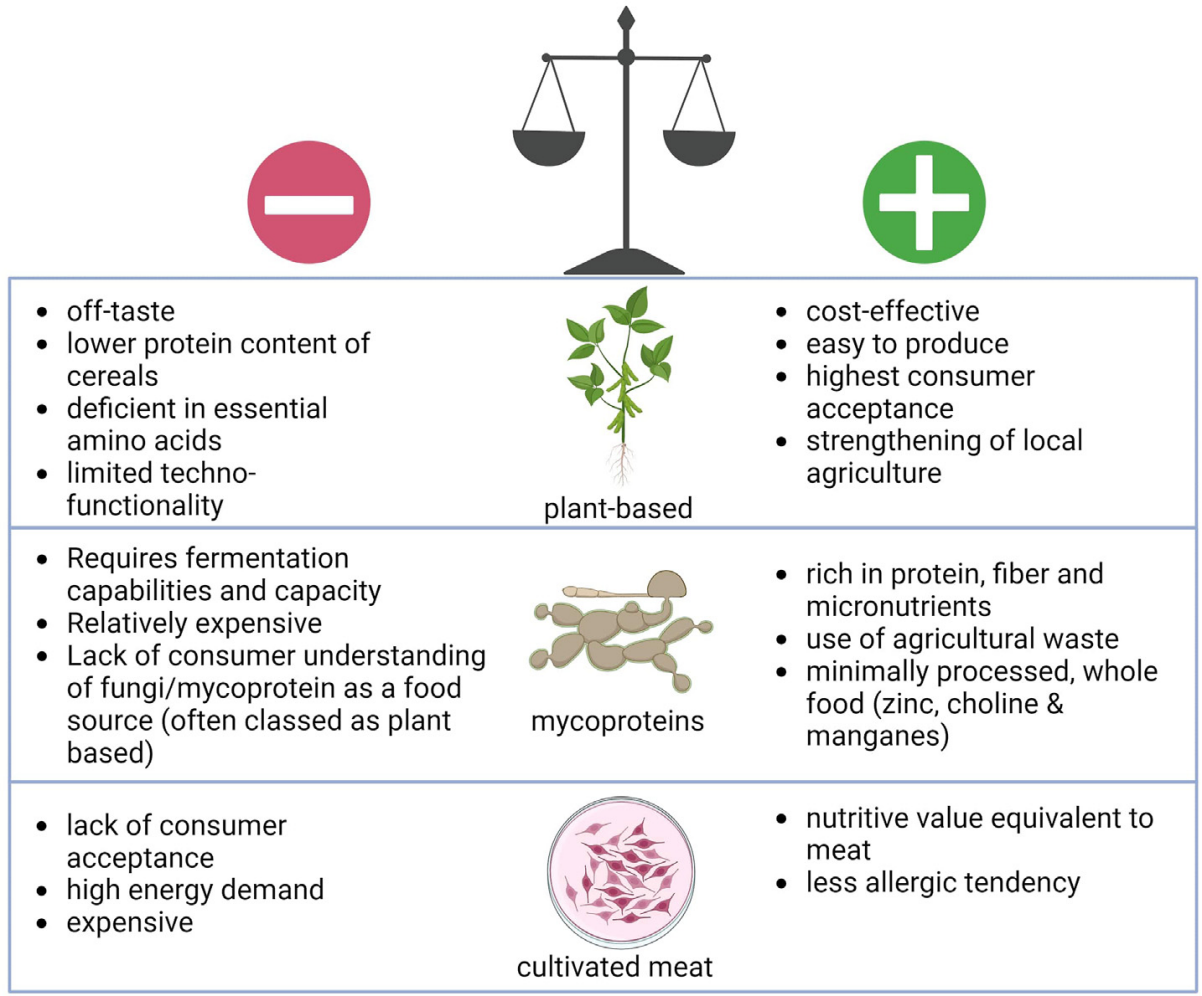
Advantages and disadvantages of the 3 alternative protein technologies in low- and middle-income countries.

Given the numerous pros and cons of traditional and novel AP from plant, fungal, and cultivated sources (Figure 1), increasing the amounts of AP in the daily diet will support the growing population, especially in LMIC, where protein malnutrition is prevalent [[1], [2], [3]]. Soy protein represents a high-quality plant-sourced, extensively studied AP that can help LMIC reach the recommended daily protein intake. Enzymatic hydrolysis and targeted fermentation may improve not only the technological and sensory qualities but also the nutritional value associated with the well-being of plant-based product consumption [12]. To increase consumer acceptance, sufficient mixing of plant-based protein with other protein sources can further improve the nutritional, sensory, and technofunctional properties. Furthermore, including diverse plant-based sources will play a significant role in meeting the protein demands. The overall aim should be to achieve greater inclusion of other plant-based AP sources in the food supply to promote diet diversification and meet the daily requirement of essential amino acids.

Mycoprotein, created via sterilized fermentation, harvesting, and mixing with texturizers, exhibits low carbon and water footprints. Further research in mycoprotein fermentation technology, texture, and flavor can help increase production efficiency and consumer acceptance. Moreover, investment in mycoprotein fermentation facilities for LMIC and local production of mycoprotein is crucial for reducing production costs and creating affordable products needed to effectively establish mycoproteins in LMIC markets.

Consumer awareness, acceptance, and large-scale production of cultivated meat remain a challenge in LMIC and HIC alike. Investments in infrastructure, particularly bioreactors, are needed to establish this sector and fulfill the demands of a growing population. The optimization of growth medium, maintenance, and energy costs will play a relevant role in the final price and thus desirability and acceptability of cultivated meat and seafood among consumers. Therefore, due to low production costs, existing infrastructure, and consumer acceptance, plant-based proteins such as soy may currently be the most attractive AP to support protein intake in LMIC.

## Author contributions

The authors‘ responsibilities were as follows – KG and ASSH: conducted the literature review; KG and ASSH: wrote the first draft. HET, TJAF, WLG, BK, and VS: provided suggestions that led to subsequent drafts. VS: finalized the draft and led the submission process. All authors read and approved the final manuscript.

## Data availability

Data described in the manuscript, code book, and analytic code will be made available upon request.

## Conflict of interest

WLG voluntarily participates as the faculty advisor of the Alternative Protein Project at Johns Hopkins University, which collaborates with the Good Food Institute without funding. HT and TF work for Marlow Foods Limited, an alternative protein company, but did not receive financial incentives to contribute to this article. All other authors have no conflict of interest to declare.

## Acknowledgments

We thank Neha Tripathi and Seoyoon Choi from the Johns Hopkins University for sharing ideas on this topic and Klaus Kraemer, Kesso G. van Zutphen-Kuffer, Jimena Monroy-Gomez, and Jacquelyn R. Bedsaul from *Sight and Life* Foundation for their critical review and coordinating and funding this special supplement.
